# The Cholera

**Published:** 1871-11

**Authors:** 


### THE CHOLERA

Cannot become general in the Northern States after the first of September; we need have no fear whatever of its coming or its general prevalence, until after the first of July of 1872.

This disease has always arisen in the jungles of India, and has travelled steadily westward until it reached our eastern shores, to pass across our country and be lost in the great Pacific Sea.

Cholera makes its way along the great lines of travel, whether north, south, east or west, and as within a few years past the time of circumnavigation has been reduced to months instead of years, its passage around the world will be facilitated accordingly. But hot weather is essential to its prevalence, and if next summer is a cool summer, it is an absolute impossibility that it should exist here in the North to any appreciable extent, — only a few scattering cases.

Preventives taken internally will have a tendency to increase the liability to an attack.

There is only one infallible preventive of epidemic cholera known, and that is always, everywhere, and infallibly efficacious; it is

ABSOLUTE CLEANLINESS

In person, in skin, in scalp, in clothing, in chamber, in parlor, in kitchen, in cellar, in the back yard and in the front yard, in closet, sink, and gutter, and street.

If the cholera does come, the ignoramus and the knave will be flouting his lying cholera preventives and infallible cures, by handbills, circular, and newspaper; but all these are not only useless, — they are absolutely ruinous, because they will have the effect to make us rely upon them to the exclusion of the only safe plan.

1. Perfect cleanliness.
2. Regular eating.
3. Mental composure.

4. Deliberate exercise and labor.
5. Maintaining the daily bodily functions.
6. Making no great or sudden changes in eating, drinking, work, or sleep.

### ESSENCE OF CHOLERA.

The very essence of cholera is a thin, light-colored, watery discharge, without pain, and more than once in twenty-four hours ; remember, thin, light-colored, frequent, without pain, leaving the person in a quiet, placid condition, with one overshadowing desire, not to be required to budge an atom.

The first step towards cure in every case, and under all circumstances, is to go to bed ; the second, send for a doctor ; don't make a fool of yourself by attempting to be your own physician.

### IS CHOLERA CATCHING ?

If in very warm weather, or in a very warm room, a person breathes into his lungs, or swallows into his stomach with the saliva, any portion of that which has come to-day or months ago, in its dry or in its moist state, from the bowels of a person who has cholera, then cholera may be taken.

Dr. Marsden, an eminent physician of Quebec, and who has written with more personal knowledge of the subject than any other man in America, and whose high position in the New Dominion and among our eminent men is demonstrative that any statement he makes is a guarantee of its truth, has published several cases, showing most undeniably that the handling of a mattress, weeks after a man had died of it, gave an undertaker the cholera in Quebec, of which he died; that persons died of cholera who opened a trunk in mid ocean, which trunk contained the clothing of a person who had died months before of cholera, in the interior of Europe; showing that clothing carries cholera, and that the thing carried floats in the air, and is conveyed into the body by breathing and swallowing. Yet persons may have such vigorous health, may be so cleanly, may live such regular, quiet, and sedate lives, as to be sure to resist these influences. It is well that the people be thinking of these things, and endeavor to inform themselves, before the disease comes upon them like an avalanche. A calm and intelligent view of the subject is itself a preventive to a very considerable extent.

